# Giant Uterine Leiomyoma Causing Severe Hemodynamic Instability

**DOI:** 10.1097/og9.0000000000000059

**Published:** 2025-02-06

**Authors:** Saed Almasarweh, Paul Buderath, Peter Rusch, Marc Ingenwerth, Rainer Kimmig

**Affiliations:** Department of Obstetrics and Gynaecology and the Department of Pathology, Essen University Hospital, Essen, and the Department of Obstetrics and Gynaecology, Augusta-Hospital Bochum-Mitte, Bochum, Germany.

## Abstract

**BACKGROUND:**

Uterine leiomyomas occasionally manifest as giant leiomyomas, displaying exceptional sizes within the uterus and abdominal cavity. These massive growths, although rare because of increased patient awareness, can severely distort pelvic structures and pose life-threatening complications by compressing adjacent organs. We report a case of a patient who presented with severe hemodynamic instability, cardiac arrest, and multiorgan failure caused by a giant 54.8-kg uterine leiomyoma, representing the heaviest weight ever reported in the literature in surviving patients so far.

**CASE:**

This article documents a case of a 56-year-old woman with a giant uterine leiomyoma in critical condition characterized by multiorgan failure and acute kidney injury necessitating urgent surgical intervention. A laparotomy and subsequent total abdominal hysterectomy were performed. Histopathologic examination revealed a giant uterine leiomyoma. Postoperatively, the patient underwent intensive care management followed by rehabilitation, exhibiting significant clinical recovery.

**CONCLUSION:**

Managing giant uterine leiomyomas demands a multidisciplinary approach, emphasizing tailored treatment strategies and specialized surgical expertise. The case report describes one of the largest leiomyomas in the literature, underscoring its rarity, and highlights the significance of timely diagnosis and appropriate interventions.

## Abstract 2

A 54.8-kg giant leiomyoma necessitating urgent surgical and medical intervention to overcome multiorgan failure highlights the importance of early management of growing uterine leiomyomas.

**Teaching Points**

- A multidisciplinary approach is crucial to effectively strategize treatment plans, manage complex cases, and address the various challenges that arise from massive tumors.
- Preoperative optimization and risk management include addressing severe comorbidities such as renal failure and cardiac complications to reduce morbidity and mortality.
- Preparedness for complex surgical scenarios includes massive intraabdominal pressure, distortion of pelvic anatomy, significant blood loss, need for extensive adhesiolysis, and potential organ compression.

Uterine leiomyomas, known as fibroids or myomas, are noncancerous growths of the uterus that often appear during the reproductive years.^1^ Giant uterine leiomyomas represent an extreme manifestation of a very common gynecologic condition, showcasing the remarkable potential for these benign tumors to reach unprecedented sizes within the uterus. Although leiomyomas are generally characterized by their variability in size, giant leiomyomas stand out for their exceptional dimensions and weight (more than 11.4 kg), often causing substantial distortion of the uterus and adjacent pelvic structures and possibly causing severe compression of abdominal organs, therefore compromising vital functions.^2^

This case report explores the case of the perioperative management and removal of a giant uterine leiomyoma weighing 54.8 kg. To the best of our knowledge, this is the heaviest uterine leiomyoma removed from a living patient with a successful postoperative outcome.

## CASE

The 56-year-old patient presented from a neighboring hospital for further treatment of a large pelvic mass. The pelvic mass had been progressing for several years but grew to a massive size 2 years previously. Further investigations were not carried out because of the patient's fear of the possible diagnosis. At the neighboring hospital, the patient presented acutely because of progressive general deterioration with severe dyspnea and circulatory decompensation. The initial examination revealed an acute kidney injury with severe hyperkalemia (8.0 mmol/L), anemia with a hemoglobin of 4.3 mg/dL, and drastically elevated inflammatory parameters. The patient immediately received 4 units of packed red-blood cells and underwent dialysis to alleviate the hyperkalemia and acute renal injury. A native computed tomography of the thorax and abdomen revealed a massive abdominal cystic structure, which radiologists suspected arose from one of the ovaries, that caused severe compression of abdominal organs and diaphragm. After the patient was started on vasopressors for circulatory stabilization, she was transferred to our tertiary university hospital for further diagnostics and treatment. Shortly after the patient arrived at our intensive care unit (ICU), she had an episode of asystole, which was attributed mostly to compression of the inferior vena cava during mobilization. After cardiopulmonary resuscitation for a few minutes, the patient regained sinus rhythm. The patient was then stabilized in the ICU, and the decision was made to perform an urgent explorative laparotomy because of the current unstable and further deteriorating state of the patient.

**Fig. 1.**
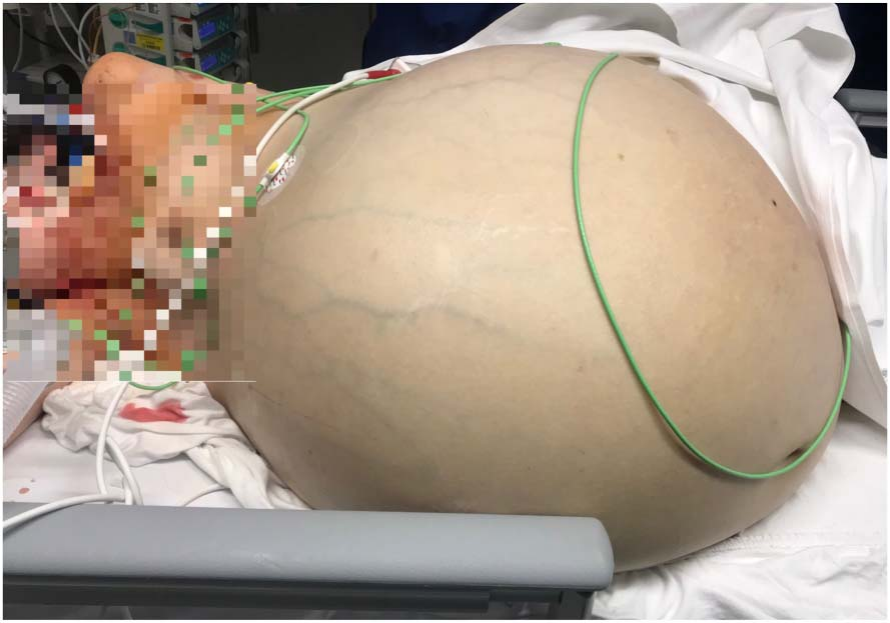
Preoperative situation of the massively distended abdomen with dilated veins. The patient was placed in the lateral recumbent position to prevent circulatory failure caused by intraabdominal pressure on the great abdominal vessels.

**Fig. 2.**
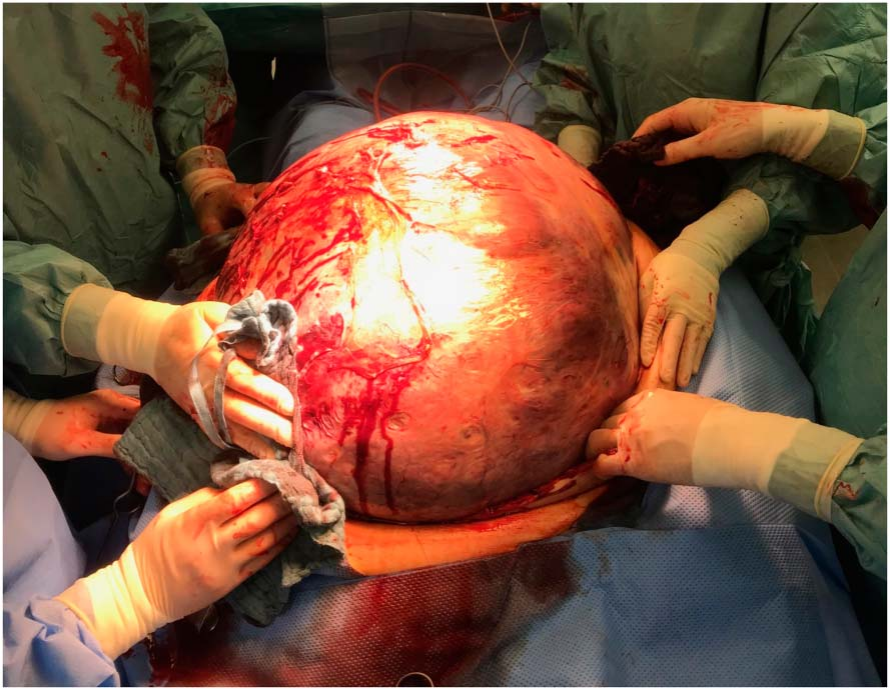
Intraoperative finding of the giant leiomyoma after the midline laparotomy was performed.

**Fig. 3.**
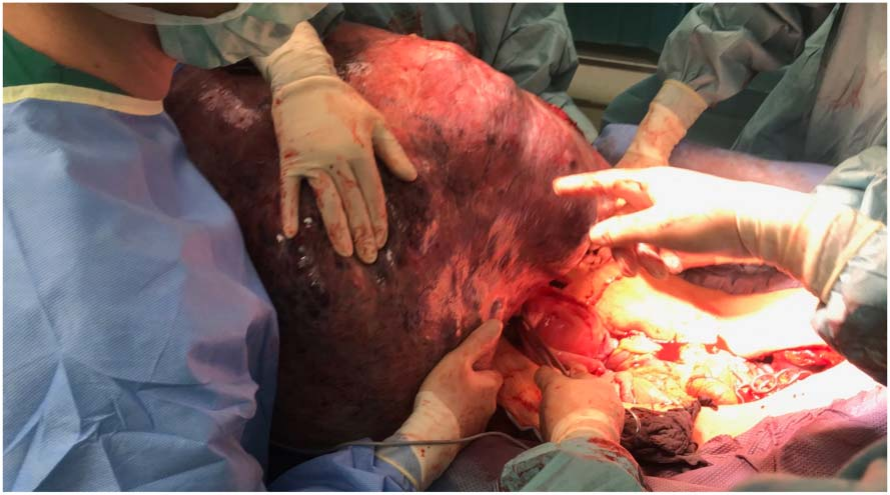
Operative field after the tumor was lifted over the symphysis pubis revealing the broad pedunculation to the uterine fundus.

The patient was stabilized by the anesthesia team and was cleared for operative therapy. The abdomen was massively enlarged, with visible dilated veins indicating increased intraabdominal pressure and a palpable tumor that reached just below the xiphoid process (Fig. 1). A median laparotomy up to the xiphoid process was performed (Fig. 2). The tumor, which filled the entire abdomen, was adherent at various points to the anterior abdominal wall and to the lateral pelvic walls. After thorough adhesiolysis, the massive tumor was then lifted over the symphysis pubis, which then revealed that the tumor was broadly pedunculated on the uterine fundus and obviously corresponded to a uterine leiomyoma rather than being, as initially suspected, an ovarian tumor (Fig. 3). The pedicle connecting the tumor to the uterus was then ligated, and the massive tumor removed. There was no evidence of peritoneal or bowel involvement in the pelvis. Because of the in situ findings, unclear tumorigenicity, and the critical condition of the patient, the decision was made to complete the procedure with a total abdominal hysterectomy with additional bilateral salpingo-oophorectomy. Cytologic assessment of ascites was performed. Postoperatively, the patient was transferred back to the ICU for further management.

*Almasarweh. Large Leiomyoma Causing Decompensation. O&G Open 2025*.

*Almasarweh. Large Leiomyoma Causing Decompensation. O&G Open 2025*.

*Almasarweh. Large Leiomyoma Causing Decompensation. O&G Open 2025*.

**Fig. 4.**
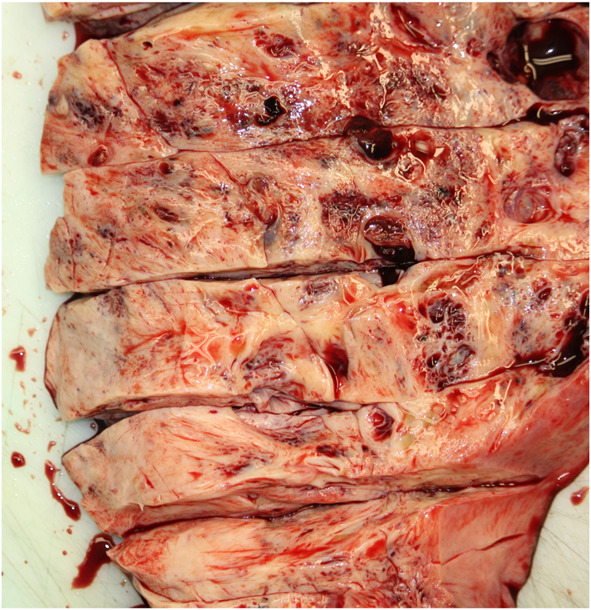
Pathologic macroscopic image of the uterine leiomyoma after slicing.

**Fig. 5.**
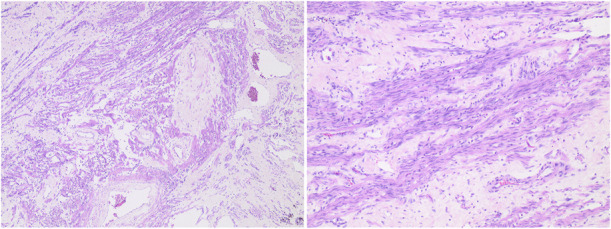
Histopathologic evaluation with hematoxylin-eosin stain revealing a benign uterine leiomyoma (original magnification left ×40, right ×200).

**Fig. 6.**
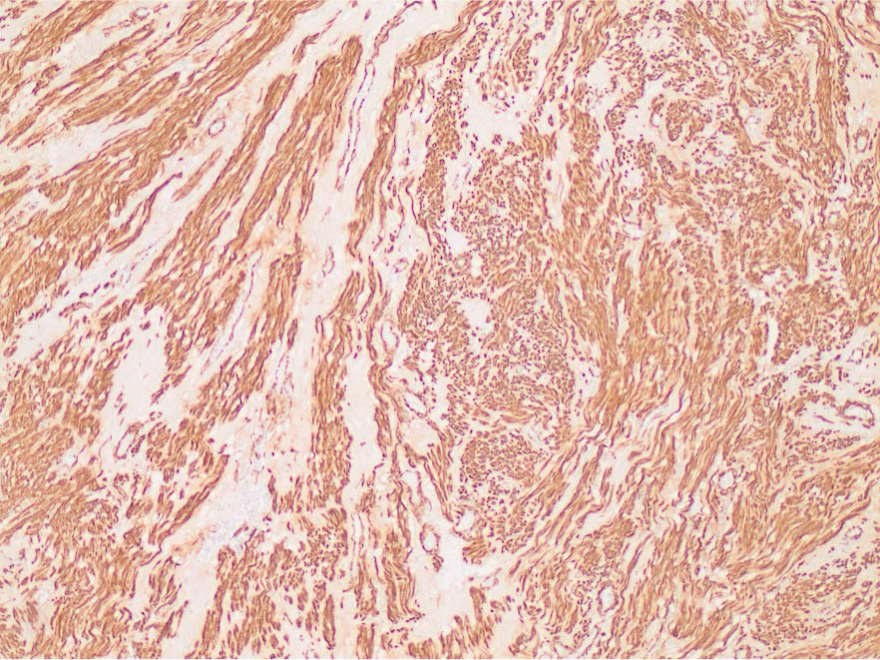
Immunohistochemistry revealing strong positivity for actin (actin stain, original magnification ×100).

Pathology confirmed a solitary huge uterine leiomyoma measuring 63×52×53 cm and weighing 54.8 kg (Fig. 4). Histologic examinations revealed a benign spindle-cell neoplasm with intersecting fascicles without nuclear atypia (Fig. 5). Mitoses were rare in the tumor cells. Degenerative changes were visualized with fibrosis and microcalcification, as well as hemorrhagic areas. Immunohistochemistry showed strong positivity for actin (Fig. 6), thus confirming the diagnosis of a conventional leiomyoma.

*Almasarweh. Large Leiomyoma Causing Decompensation. O&G Open 2025*.

*Almasarweh. Large Leiomyoma Causing Decompensation. O&G Open 2025*.

*Almasarweh. Large Leiomyoma Causing Decompensation. O&G Open 2025*.

Further tests revealed rapid deterioration of organ functions attributable to severe intraabdominal compression and vascular depletion, resulting in multiorgan failure (liver, heart, kidney, and lungs), which were treated rigorously in the ICU. On the 22nd day, the patient was then transferred to a rehabilitation facility to accelerate postoperative recovery. The patient had to slowly regain mobility and cognitive function through an 8-week rehabilitation. A 5-year follow-up revealed complete recovery except for mild heart failure, which is chronically treated with metoprolol, ramipril, ivabradine, spironolactone, and levothyroxine for hypothyroidism. Postoperatively, the patient underwent regular gynecologic checkups, which were uneventful, and regular visits to a cardiologist, with good heart function through the use of the aforementioned medications. The patient regained full mobility and is leading a medically unremarkable course.

## DISCUSSION

Uterine leiomyomas, the most common benign tumor in women of reproductive age,^1^ often present diagnostic dilemmas because of their varied manifestations and complex diagnostic pathways. Leiomyomas pose challenges in diagnosis primarily because symptoms can range widely. Although some women experience debilitating symptoms such as heavy menstrual bleeding, pelvic pain,^3^ or pressure on surrounding pelvic organs,^4^ others remain asymptomatic. This diversity in symptomatology often complicates the diagnosis, leading to delayed or overlooked identification of leiomyomas. Moreover, the effects of diagnostic dilemmas extend beyond medical uncertainties. Delayed diagnosis or misinterpretation of symptoms can affect treatment decisions and therefore increase patient morbidity. For instance, incorrect diagnosis might result in unnecessary surgeries or interventions, affecting a patient's quality of life and posing avoidable risks.

Giant uterine leiomyomas are those weighing more than 11.4 kg.^1^ Currently, with increased patient awareness, this condition is rare; most women present early, either as an incidental finding during a routine gynecologic checkup or as a result of symptoms that lead to abnormal menstruation or pelvic pressure.^3,4^ When they grow to enormous sizes, they can become life threatening, causing massive intra-abdominal pressure and therefore, in severe cases, multiorgan damage and cardiac arrest.^5,6^ A literature review by Viva et al^7^ showed that the incidence of giant uterine leiomyomas is about 60 cases worldwide in the past 50 years and 13 cases in a time span of 20 years up to 2021.^8^

Treating giant uterine leiomyomas presents significant challenges because of their massive size, intricate vascularity, and potential effects on surrounding organs. These exceptionally large growths often necessitate specialized approaches that go beyond standard treatment modalities while requiring a high level of skill and precision from the surgical team. One of the primary hurdles is the sheer mass of giant leiomyomas, which can significantly distort the pelvic anatomy, making surgical access and maneuverability more challenging. This distortion may obscure critical surgical landmarks and vital structures, increasing the risk of inadvertent damage and intraoperative blood loss during the procedure.

The management of giant leiomyomas should involve a multidisciplinary team including gynecologists, interventional radiologists, anesthesiologists, and internal medicine specialists to strategize the most appropriate treatment plan for each case. Advanced techniques such as preoperative uterine artery embolization to reduce blood flow to the leiomyoma or adjunctive measures such as gonadotropin-releasing hormone agonists to reduce the size and vascularity leiomyoma preoperatively have been explored to mitigate surgical complexities and reduce associated risks.^9,10^ A meta-analysis by Llewellyn et al^10^ showed that uterine artery embolization can be implemented as a safe supporting modality for treating giant leiomyomas but with a relatively higher risk of complications and reintervention compared with nongiant leiomyomas. In the case of our patient, the preoperative condition and severe hemodynamic instability rendered conservative and supportive treatment modalities impractical, therefore leaving emergent surgical treatment as the only viable treatment option. First, the efforts of the emergency department of the neighboring hospital in rapidly correcting deteriorating kidney function and electrolyte imbalance with dialysis before transfer were critical. Then, the anesthesia and gynecology teams worked to rapidly stabilize the patient and remove the massive leiomyoma to ease the intra-abdominal pressure.

The largest uterine leiomyoma ever reported in the literature weighed 63.3 kg and was removed at autopsy in 1888; up until this report, the heaviest uterine leiomyoma removed from a patient who survived the surgical procedure weighed 45.4 kg.^1^

A comprehensive literature search was conducted to identify relevant studies on giant uterine leiomyomas. A MEDLINE search was performed, covering articles published up until October 2024. The following search terms were used: “giant uterine leiomyoma,” “giant fibroid,” “large uterine fibroids,” and “massive uterine leiomyoma.” These terms were combined using Boolean operators to capture all relevant articles. No language restrictions were applied, and both case reports and original research articles were included. The search was limited to human studies, and articles that focused on the clinical presentation, management, and outcomes of giant uterine leiomyomas were prioritized. Reference lists of included articles were also reviewed for additional relevant studies.

According to the literature, the uterine leiomyoma reported in this case report is the largest leiomyoma by weight removed from a living patient with a successful postoperative outcome and a near-complete recovery. It is quite astonishing that, although the patient was severely hemodynamically unstable and had multiorgan failure, she survived with no major debilitating consequences and is leading an unimpaired life. The patient is currently very content with the outcome of the complicated situation and is thankful for the “second chance” provided to her.
